# Measurably recombining malaria parasites

**DOI:** 10.1016/j.pt.2022.11.002

**Published:** 2022-11-23

**Authors:** Flavia Camponovo, Caroline O Buckee, Aimee R Taylor

**Affiliations:** 1 Harvard T.H. Chan School of Public Health, Boston, USA.; 2 Institut Pasteur, Paris, France.

**Keywords:** Plasmodium, Molecular Epidemiology, Recombination, Pedigree

## Abstract

Genomic epidemiology has guided research and policy for various viral pathogens, and there has been a parallel effort towards using genomic epidemiology to combat diseases that are caused by eukaryotic pathogens, such as the malaria parasite. However, the central concept of viral genomic epidemiology, namely that of measurably mutating pathogens, does not apply easily to sexually recombining parasites. Here we introduce the related but different concept of measurably recombining malaria parasites to promote convergence around a unifying theoretical framework for malaria genomic epidemiology. Akin to viral phylodynamics, we anticipate that an inferential framework developed around recombination will help guide practical research, and thus realise the full public health potential of genomic epidemiology for malaria parasites and other sexually recombining pathogens.

## A new genomic epidemiological concept

The public health value of malaria genomic epidemiology (see Glossary) has been demonstrated in several recent studies. A few examples include studies identifying local versus imported transmission in Bangladesh [1] and Southern Africa [2], tracking the rise and spread of drug resistance in the Greater Mekong Region [3], quantifying transmission changes in Senegal [4], or informing on the feasibility of malaria elimination in Sri Lanka [5]. The public health value of malaria genomic epidemiology is recognized beyond the research community, particularly in light of the recent COVID19 pandemic [6], and in the context of malaria elimination, where it is used to identify transmission hotspots and imported cases, for example [7, 8]. In order to capitalize on advances in data generation (e.g. [9]), and efforts by country-level stakeholders to build capacity and integrate genomic epidemiology into policy and practice [7], methodological advances are needed to make best use of parasite genetic data [10].

Genomic epidemiology relies on the concept of measurably evolving pathogens [11, 12]. A population can be said to evolve measurably if differences among DNA sequences, sampled at different points in time, are statistically significant [11]. If a pathogen population is measurably evolving on epidemiologically relevant timescales, genomic data sampled from infections can be used to measure and map different aspects of disease transmission [12]. For example, epidemiological timescales may be on an individual host level, between serial infections or symptom onset, or on a host population level, between groups of infected individuals separated in space or time.

The conventional definition of a measurably evolving pathogen assumes genetic differences are generated by mutation [11, 12]. Pathogen genomic epidemiology as a field developed around fast-mutating RNA viruses because these viruses mutate so rapidly that differences among them can be detected with limited genomic data, typical of the pre-genomic era [12]. Whole genome sequencing has since enabled genomic epidemiology of some more slowly-mutating pathogens [12]; see https://nextstrain.org/pathogens. In general, malaria parasites are not counted among them, partly because the coherent inferential framework that applies to fast-mutating RNA viruses (phylodynamics) does not apply readily to malaria parasites since they sexually recombine.

In this article we compare the genetic consequences of recombination versus mutation in the context of malaria genomic epidemiology, examine methodological gaps, and propose an approach towards an unifying inferential framework, something akin to phylodynamics in viral genomic epidemiology. Although we focus on *Plasmodium*, the concepts apply to a broader range of sexually recombining pathogens.

## Malaria parasites mutate and sexually recombine

Both mutation and recombination generate genetic variation [12, 11]: mutation creates differences, while recombination creates new combinations of those differences. Mutational differences, *δ*, can be modelled simply as linear function of time *t*, the rate of mutation per locus per time *μ*, and the number of loci *l* : *δ* = *μlt* [12]. Sexual recombination also depends on some fixed parameters (crossover rate, number of loci, chromosomes and meioses); however, it is only “effective” when genetically distinct individuals recombine (effective recombination). Therefore, to model recombinational differences, one must also consider the external processes that bring individuals together (mating system), the amount of pre-existing variation among those individuals (population diversity) and how this variation is distributed (population structure). Malaria parasites are eukaryotes and mutate at a typical eukaryotic rate, which is slow compared to other pathogens. They recombine sexually every life-cycle, but the effectiveness of recombination can range from one, when completely unrelated parasites recombine, to zero, when clones recombine, a plausible event even in diverse populations. Moreover, the effectiveness of recombination depends on the processes that unite genetically distinct parasites: coinfection and/or superinfection with genetically distinct parasites. In this section, we discuss how and when malaria parasites mutate measurably and recombine effectively on an epidemiologically relevant timescale. We also discuss the known and unknown aspects of the processes that shape effective recombination. We focus on *Plasmodium falciparum* and *Plasmodium vivax*, the two malaria parasite species most frequently responsible for human malaria [13].

### Mutation

Malaria parasites are single-celled and, throughout the human stage of their lifecycle, haploid. Compared with viral pathogens, they have larger genomes but slower mutation rates: *P. falciparum* has a 23 megabase nuclear genome [14] and a SNP mutation rate on the order of 10^−10^ mutations per base pair per asexual generation (48 hours) [15].

Although this process generates many mutations - given the vast amount of parasites within a single malaria infection - the majority of those mutations occur singularly and are purged [9]. A well-defined core genome is often used for *P. falciparum* genomic epidemiology [16]. Among a population of infecting parasites, it accrues an estimated 0.84±1.8 non-purged mutations per month [17]. That value increases to 2.92±2.3 non-purged mutations per month (comparable to measurably mutating viruses) when advanced technologies are used to extend the accessible region of the genome [17]. Thus, with a generation interval of around 3 months for *P. falciparum* [18], it is theoretically possible to differentiate malaria parasites along a transmission chain using mutation. However, those mutations are only identifiable when parasites from different infections do not recombine, e.g. in near-elimination settings where transmission is extremely low and clonal propagation is extensive [17].

### Recombination

While malaria parasites might accrue a small number of non-purged mutations over the course of one lifecycle, 50% of the genome is expected to differ if recombination with an unrelated parasite occurs. This means that recombination has greater potential to generate measurable variation on epidemiologically relevant timescales. This potential has been demonstrated by various studies (e.g. [19, 20, 21]), using either identity-by-descent (IBD) as a measure of recombinational relatedness or identity-by-state (IBS), a correlate of IBD [22].

Recombination is obligate in the malaria parasite lifecycle. Human blood stage parasites differentiate into gametocytes that are imbibed by the mosquito, where they differentiate promptly into gametes, and pair to sexually recombine approximately three hours after ingestion [23], each pair resulting in an oocyst, with usually <5 oocysts per mosquito [24]. The speed of fertilization impedes recombination between parasites from different blood meals and thus different people, unless a mosquito feeds on different people in very quick succession - a phenomena that likely does not contribute significantly to malaria epidemiology. *P. falciparum* gametes are estimated to crossover with probability 7.4 × 10^−7^ per base pair [16]. This implies, on average, 0.01 crossovers per 13500 base pairs and approximately one crossover per chromosome, of which *P. falciparum* has 14. This means that after recombination between unrelated parasites, we expect offspring to be 50% related to their parents with, on average, one contiguous IBD segment per chromosome. Even without crossovers, sexual reproduction can generate variation because offspring inherit a random combination of their parental chromosomes.

### Effective recombination

**Figure 1:**
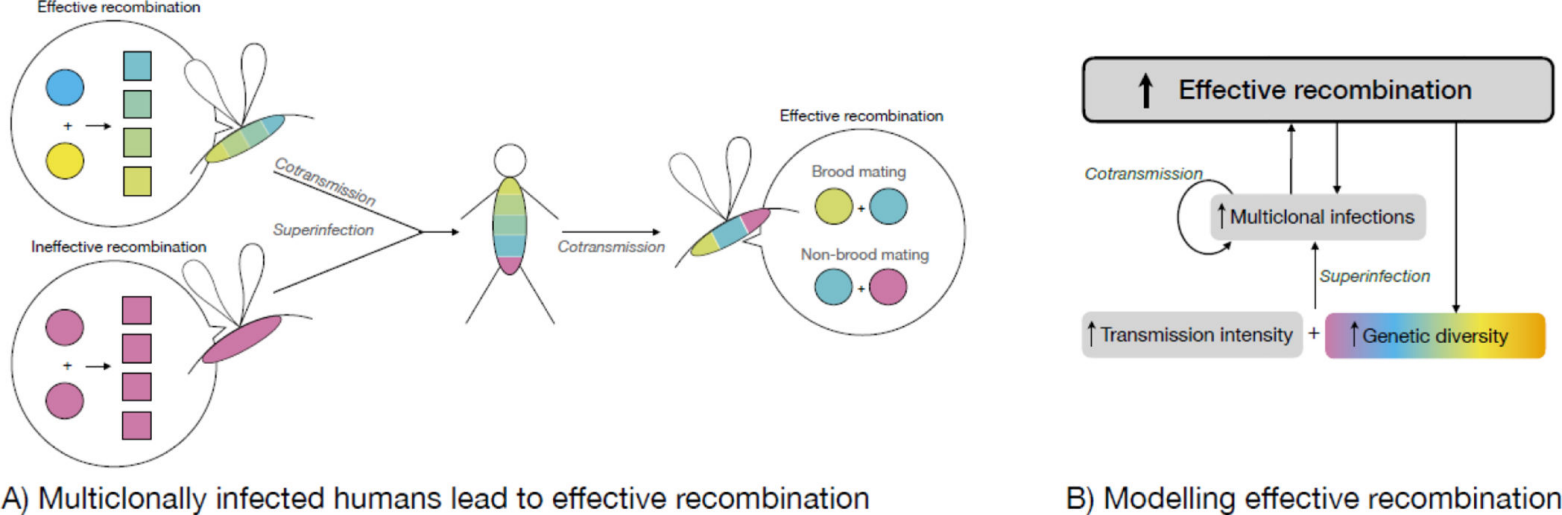
Effective recombination. (A) Human-to-mosquito cotransmission leads to effective recombination but its effectiveness depends on the type of mating and thus the processes that generated the multiclonal human infection (cotransmission and superinfection). Different colours represent genetically distinct parasites within infections (host fill) and during recombination (parental gametes, circles; offspring, squares). Brood mating following mosquito-to-human cotransmission is more likely to have low effectiveness due to probable inbreeding, whereas non-brood mating following superinfection is likely to have high effectiveness due to probable outbreeding if the population is largely outbred. (B) The frequency of occurrence of effective recombination is expected to increase with higher transmission intensity. Given genomic diversity in the population, greater transmission results in more superinfections, leading to more multiclonal infections in humans and mosquitoes, which in turn increase the opportunity for cotransmission. Multiclonal infections allow for effective recombination, which in turn gives rise to more multiclonal infections and greater genomic diversity. The effectiveness of recombination (not shown) depends on the routes via which multiclonally infected mosquitoes are generated: more routes via superinfection will lead to more non-brood mating, while more routes via mosquito-to-human cotransmission will lead to more brood mating (panel A).

Although recombination is obligate, it is not always effective. Malaria parasites can self, i.e. genetically identical parasites can recombine, in which case recombination is ineffective. Selfing is inevitable when a mosquito feeds on a single monoclonal infection. Otherwise, selfing, inbreeding and/or outcrossing can occur, where inbreeding refers to partially effective recombination between related parasites, outbreeding refers to fully effective recombination between unrelated parasites, and the occurrence of one or more events depends on the number of parasite pairs that recombine. The extent of effectiveness depends principally on the composition of multiclonal human infections on which mosquitoes feeds (Figure 1A).

A multiclonal human infection can be generated in two, non-mutually exclusive ways: by a single mosquito bite transmitting genetically distinct parasites (cotransmission) and/or by several mosquito bites (superinfection), e.g. Figure 1A. Parasites from different mosquitoes cannot belong to the same brood. Within the mosquito, parasites can belong to the same brood, which can contain clones, strangers, and siblings [25, 26]. Inter-brood relatedness of parasites depends on the diversity and structure of the parasite population, and intra-brood relatedness depends on the relative occurrence of clones, strangers, and siblings within the brood and on the relatedness of the parental gametes. This means that the level of effective recombination between parasite genotypes depends on the relative frequency of both brood and non-brood mating between parasites, which, in turn, depends on cotransmission and superinfection between hosts (Figure 1A). That is to say, malaria parasites are not panmitic and the generation of diversity is linked to transmission intensity in a non-trivial way.

Both cotransmission and superinfection are expected to increase with transmission intensity, thereby increasing the overall prevalence of multiclonal infections (as has been observed inversely [27]) and the frequency of occurrence of effective recombination (Figure 1B). It is more difficult to predict how the effectiveness of recombination will be impacted by transmission intensity: given pre-exisiting variation, more infectious bites lead to a higher rate of superinfection and thus more opportunities for non-brood mating, which leads to outcrossing if the population is diverse and largely unstructured. However, more outcrossing leads to more mosquito-to-human cotransmission of outbred offspring that can consequently brood mate, which almost certainly leads to some inbreeding. As such, although superinfection and outcrossing both lead to effective recombination (Figure 1B), superinfection likely amplifies its effectiveness while mosquito-to-human cotransmission likely attenuates it (not shown).

Observations from field studies testify to the complexity of this system, using descriptive statistics of parasite genetic data as proxy indicators. Generally, high estimates of diversity and average multiplicity of infection (MOI) suggest high transmission, while evidence of prevalent clonal clusters and monoclonal infections suggest low transmission [28]. However, this relationship is sometimes unclear [29], especially in the presence of gene flow [30, 5, 31, 32, 33, 34, 35]. Moreover, interpretation is hampered by extensive spatial heterogeneity [36], which is accentuated as transmission declines [37, 38], but does exist in high transmission [39, 40], though it is harder to detect [41]. Relapses add additional complexity for *P. vivax*, where MOIs can reflect present or past innoculations and thus are generally higher that those of *P. falciparum* [42].

For either species, what these processes collectively mean for the effectiveness of recombination is unclear: in low transmission settings, evidence of high *P. vivax* population diversity and average MOI has been observed together with significant linkage disequilibrium (*LD*, indicative of low effective recombination) [5, 37]; while in similarly low transmission settings, evidence of low *P. falciparum* population diversity and prevalent monoclonal infections has been observed together with low LD [43]. In high transmission settings, evidence of *P. falciparum* inbreeding persists [44, 45], consistent with the expected effect of brood-mating, and in both low and high transmission settings, *P. falciparum* multiclonal infections contain highly related parasites [46, 26, 47].

To summarise, effective recombination has greater potential than mutation to generate variation that is measurable on an epidemiological scale, but, unlike mutation, its effectiveness is inextricably linked to the epidemiological context in a complicated way (Figure 1B). Although some models of the mosquito stage of this highly complex system exist [48, 25, 26], its entirety is not understood well enough to translate into a functional form (see Outstanding Questions).

## Malaria genomic epidemiology at present

**Figure 2:**
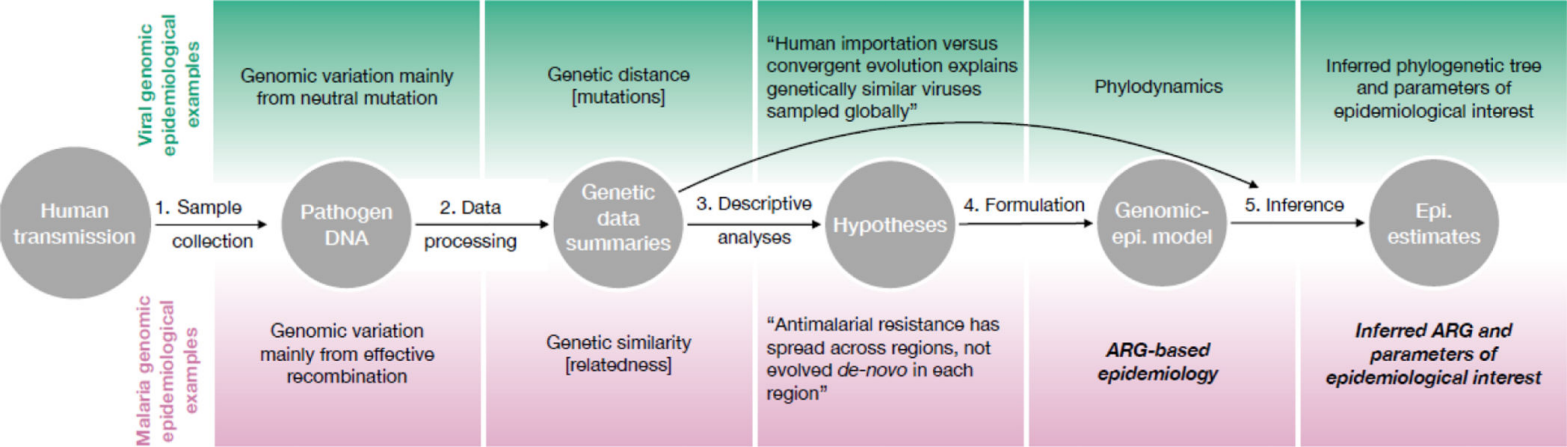
Possible series of genomic steps in pathogen genomic epidemiological studies. First, genomic data are collected. To be useful, those data must contain variation that has accumulated on an epidemiological relevant scale, e.g. due to mutation or due to recombination, processes that data summaries generated in step two often reflect. Step three involves a suite of descriptive analyses; for example, the computation of descriptive statistics of pathogen diversity and differentiation, population assignment and clustering analyses. A model that connects the genomic processes to epidemiological ones is formulated in the forth step, typically using mathematics to articulate hypotheses concretely. This process incites clarification and thus is valuable in and of itself. An arrow connects data to the fifth step, because data are used to infer the parameters of the model. This step also links to epidemiological data; however, these are not shown. Above and below the steps, examples are provided for viral and malaria genomic epidemiology, respectively. Those that do not currently exist are highlighted in italic and bold.

For the practical application of pathogen genomic epidemiology, data should be used to infer parameters of epidemiological interest under a cohesive statistical model that links the processes that generate the genetic data to epidemiological ones (e.g. in viral genomic epidemiology, phylogenetic models are linked to coalescent or birth-deaths models in a framework called phylodynamics [49, 50, 51, 52]). Under a statistical model, interpretation is straightforward (phenomena of interest can be expressed explicitly as parameters and their dependence on hypothesised predictors evaluated [53]), as is prospective study design (e.g. using posterior predictive simulation or by maximizing the Fisher information of parameters of interest, as in [54]). Various steps build up to this model (Figure 2). Typically, malaria genomic epidemiological projects culminate in hypotheses generated by descriptive analyses (step three of Figure 2) because a cohesive inferential framework is lacking.

Descriptive analyses in malaria genomic epidemiology are related to those across malaria genomics more generally (see Box 1). They generate valuable hypotheses but they are also liable to generate some spurious associations. Moreover, descriptive analyses cannot provide conclusive answers to the questions malaria genomic epidemiology ultimately seeks to answer [55]. For example, a clustering analysis might reveal population structure that suggests gene flow to a region is restricted [56] and thus that the region is a suitable candidate for targeted intervention, but without a model under which this hypothesis can be falsified, one cannot reject competing processes, such as drug selection.

Because of recombination, phylodynamic frameworks cannot be applied directly to malaria (phylodynamic methods that accommodate recombination treat it as noise and not signal [12]) and an equivalent framework for malaria is lacking. However, efforts to develop simulation-based models are ongoing (e.g. the R package SIMPLEGEN, https://mrc-ide.github.io/SIMPLEGEN/). Agent-based models linked to genomic processes have been used to estimate *R*_0_ and changes in transmission intensity [57, 4], to investigate the relationship between different descriptive statistics of parasite genetic data and transmission intensity [58, 55], to study the effect of heterogeneity on the spatial distribution of multiclonal infections and on the stability of transmission [38], and to study the effect of selective pressures on evolution under different transmission settings [59]. These models are used to simulate data under arbitrarily complex scenarios whose parameters are known. They are thus very versatile, but at a cost: in general, they are too complex for full statistical inference. However, they can be calibrated by comparing model predictions to real data, and then used to design prospective studies.

## The future of malaria genomic epidemiology

The ultimate unifying inferential framework for malaria genomic epidemiology would centre around ancestral recombination graphs (ARGs), in the same way phylodynamics centers around phylogenetic trees. In the case of malaria, the mating system would link host-level parameters of epidemiological interest to the parasite genomes that feature in the ARG.

**Figure 3:**
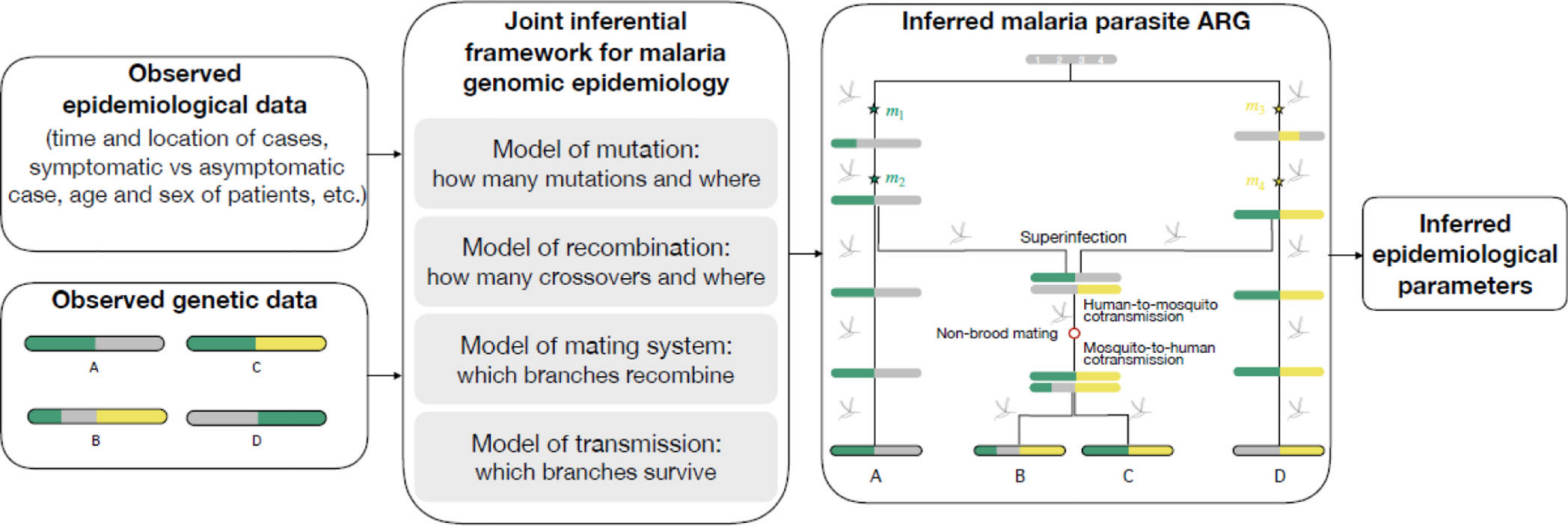
Overview of ARG-based malaria genomic epidemiology. The goal of genomic epidemiology is to use observed genomic and epidemiological data (left) to infer epidemiologically relevant parameters (right). One way to do this would be to formulate a model around the malaria parasite ARG. In this example, four genetically distinct DNA segments (A, B, C, D) each of length equal to four loci (indexed by *i* ) are linked back to a recent common ancestor (grey) via four per-locus mutations (*m*_*i*_ ) and one effective recombination event (red circle) over five generations. The observed sequences are outlined in black whereas unobserved inferred sequences deeper within the ARG are not. This example only depicts sequences sampled from the human host and thus some sequences present in the mosquito are omitted; specifically, two of the four haploid meiotic products that were produced after effective recombination.

An ARG is a graph that links DNA sequences by both mutation and effective recombination. It can also be viewed as a sequence of phylogenetic trees, one tree for each locus along the genome, where trees from one loci to the next are transformed if effective recombination events occur between the loci [60]. It is a summary of all the coalescence and effective recombination events in the genealogical history of a set of nucleotide sequences and thus very powerful [60, 61]. Moreover, if inferred under a framework that includes an epidemiological model whose parameters can be expressed as a function of parasite ancestry, an inferred ARG leads to estimates of epidemiological interest (Figure 3). ARG-based malaria genomic epidemiology does not exist yet in large part because ARG inference is expensive both computationally and operationally [60, 62, 63], but human population genetic studies are advancing ARG inferential methods [61]. ARG-based methods require adjustment for malaria populations, specifically to account for dynamic rates of selfing, inbreeding and outbreeding. In particular, an advanced ARG-based model is needed since the effectiveness of recombination is an emergent property of the parasite mating system, and a fixed estimate or average rate (as for selfing in [64]) would not represent underlying transmission, which is ultimately the target of inference.

The locations of effective recombination events in a malaria parasite ARG and thus the effectiveness of recombination are governed by the parasite mating system: branches are sampled uniformly at random among branches allocated to different broods when non-brood mating occurs, branches are sampled among those allocated to the same brood when brood mating occurs; otherwise, when recombination is not effective, branches are propagated from one generation to the next. How to model the malaria parasite mating system is a difficult open question but models from population ecology provide some inspiration. The use of IBD, though relatively new to malaria genomic epidemiology, is not new to population ecological studies of eukaryotes, where sexual recombination is the primary source of genomic variation. For example, Close Kin Mark Recapture, a method recently developed to estimate time-series of adult population size and survival of fish or other species [54], such as mosquitoes [65], defines priors for kinship probabilities using demographic models with parameters such as the adult population size, birth rate, and individual survival probability, while accounting for possible covariates including date and location of capture. Theoretically, this framework could be adapted to malaria, where a transmission model would replace the demographic model with epidemiological covariates (e.g. case-specific characteristics) that modify the probability of kinship among malaria parasites.

## Concluding Remarks

Malaria genomic epidemiology is an exciting field of research which has proven useful for informing malaria surveillance and in which interest is growing among malaria control programs and policy makers. However, it is largely dominated by descriptive genomic data analysis that are disconnected from routine epidemiological analyses and are frequently retrospective. Although insightful, these types of analyses lack a clear common framework and are limited to speculative interpretation of the underlying transmission dynamics. We introduce the concept of measurably recombining malaria parasites in the hope that it will encourage development around a unifying inferential framework under which models can be developed and thus used for hypothesis-driven analyses and statistically robust prospective study design. This is by no means an easy task (see Outstanding Questions) but ongoing progress towards it will advance malaria genomic epidemiology thereby helping to promote its full potential for public health.

## Highlights

- The recent pandemic has further highlighted the public health potential of infectious disease genomic epidemiology.
- For viruses, epidemiological parameters can be estimated under powerful phylodynamic models using both epidemiological and genomic data jointly. An equivalent framework for malaria parasites is lacking because they recombine.
- Recombination between malaria parasites can generate epidemiologically relevant variation, but recombination is sometimes ineffective, depending dynamically on transmission. This makes it hard to model. It also means it could link epidemiological and genomic processes if they were modelled jointly.
- Given the potential of recombination, efforts to build a unifying inferential framework around the malaria parasite ancestral recombination graph (ARG) are merited. ARG-based genomic epidemiology could someday be an equivalent of phylodynamics.

## Box 1: Malaria genomic epidemiology in context

Pathogen genomic analyses use different sources of variation that cover different spatio-temporal scales, not all of which are suited to epidemiology. Figure I provides an overview, to which there are exceptions, e.g. [17].

Mutation is the main source of genomic variation used in viral phylodynamic studies, which range from intra-host to inter-continental scales. Examples of phylodynamic studies of RNA-viruses across different scales include a retrospective study of city-scale spatial spread of Influenza A/H3N2 [66]; the investigation of the 2014 Ebola outbreak in Sierra Leone [67]; and tracking the SARS-CoV-2 pandemic (reviewed in [68]).

Mutation is also the main source of genomic variation used in malaria phylogenetic studies, which often explore species origin on a large spatial scale [69]. These studies often use genomic data where the assumption of no recombination holds (e.g. genomic data from different parasite species, or DNA data on non-recombining mitochondria).

Analyses of allele frequencies within and across populations feature in many population genetic analyses of malaria parasites. Allele frequencies change with selection (e.g. from drug pressure), gene-flow (e.g. due to the mobility of infected hosts) or genetic drift (particularly in small populations), typically at a rate slower than that which is epidemiologically relevant on an individual level [22, 70]. That said, in small populations with limited effective recombination, allele frequencies may vary at a rate equivalent to recombination (e.g. [71]). In any case, descriptive statistics of allele frequency variation (population-level) provide less resolution that those of recombination-based metrics (individual-level).

Recombination-based metrics (e.g. summaries of IBD along the genome and relatedness, which averages over the genome) are popular when studying signals of selection, malaria parasite population connectivity and population structure (e.g. [20, 72, 2, 1]). To characterise population structure, IBD- and IBS-based similarity matrices are often input into clustering algorithms, which include tree-like algorithms but are not strictly phylogenetic models [73]. Unlike allele frequencies, which may or may not change over a short period of time, parasites always recombine between different human hosts. Therefore, recombination-based metrics have the potential to vary between individuals, providing recombination is effective.

Multiplicities of infection vary between infected hosts. They do not require any change on the parasite level. Otherwise stated, variation on the parasite level might be fixed, but partitioned differently among hosts. As such, among all genetic metrics, estimates of the multiclonal infection prevalence and MOIs respond most quickly to changes in transmission [27, 55, 58]. They can also change between initial and recurrent infections within individuals.

## Outstanding questions

1. How does transmission impact the effectiveness of malaria parasite recombination? And how do these processes translate into a model of the malaria parasite mating system? More specifically,
  - How does transmission translate into the frequency of occurrence of cotransmission and superinfection between hosts, and thus brood and non-brood mating between parasites.
  - For a given transmission setting at equilibrium, how does brood and non-brood mating between parasites translate into outcrossing, inbreeding, and selfing between parasite genotypes.
  - For a given transmission setting at equilibrium, what is a representative distribution of stranger, sibling, and clonal parasites within a brood.
  - For a given transmission setting at equilibrium, what are the representative distributions of relatedness and IBD between stranger, sibling, and clonal parasites within a brood, and between stranger parasites from different broods.
  - How do the underlying processes translate into measurable correlates of transmission (e.g. the prevalence of human infection and the entomological inoculation rate) and measurable correlates of effective recombination (e.g. measures of genetic diversity, linkage disequilibrium, and IBD).
2. Can statistical inference be improved for simulation models in malaria genomic epidemiology.
3. Can a computationally efficient joint inference framework be developed for ARG-based genomic epidemiology?
4. Could an ARG-based epidemiological framework be adapted to the malaria parasite mating system? Specifically, dynamic rates of selfing, inbreeding and outbreeding that link to epidemiological processes.

## Glossary

ancestral recombination graphs: Graphs that link DNA sequences by both mutation and effective recombination.
brood: Used herein to refer to a collection of parasites produced when one or more oocysts hatch collectively in a mosquito. Note that because of the speed of fertilisation, parasites ingested from different humans in a superinfected mosquito likely do not have an opportunity to mate and likely have staggered hatchings. As such parasites from a superinfected mosquito can either belong to the same or different broods.
brood and non-brood mating: Used herein to refer to mating between parasites from the same and different broods, respectively. Brood mating is comparable to non-random mating in population genetics more generally, since mates are not sampled uniformly from the population at large. We avoid the term non-random mating, however, because it could be misconstrued: when parasites brood mate, they are sampled randomly, but from the brood.
cotransmission: The transmission of genetically distinct parasites from mosquito to human or vice versa upon a single mosquito bite.
effective recombination: Used herein to refer to recombination between genetically distinct individuals. The effectiveness of recombination can range from low (inbreeding - recombination between genetically distinct but related individuals) to high (outcrossing - recombination between genetically distinct and unrelated individuals). When genetically identical individuals recombine (selfing), recombination is ineffective.
generation interval: The time between infection onset in consecutive human hosts in the transmission chain.
genomic epidemiology: Using genomics to study disease determinants in epidemiology. Used herein to refer to the use of pathogen genomic data to track pathogen populations in space and time for public health purposes, as opposed to the study of human genetic determinants of noninfectious diseases.
identity-by-descent: IBD; two alleles are identical-by-descent (IBD) if they are both copies of an ancestral allele; a chromosomal segment is IBD if it is descended intact (unbroken by recombination) from a common ancestor.
identity-by-state: IBS; two alleles are identical-by-state (IBS) if they are biochemically alike, e.g. both adenine, regardless of whether they are identical-by-decent or identical-by-chance.
multiplicity of infection: MOI; also referred to as complexity of infection, COI; the number of genetically distinct parasite genotypes within an infection, where genotype is used here to refer to a specific example of the malaria parasite genome.
phylodynamics: Models linking phylogenetic and epidemiological processes in order to estimate epidemiologically relevant parameters.
phylogenetic models: Also referred to phylogenomics. The study of phylogeny, which is the evolutionary history between groups of organisms according to a taxon rank (such as genus, species, or strains). Molecular phylogeny uses nucleotide sequences to reconstruct phylogenetic trees.
superinfection: The transmission of parasites to an already infected human or mosquito, via infectious bites from multiple mosquitoes to one human (or via a single mosquito biting multiple infectious people, respectively.
